# Can We Identify Sources of Fine Particles Responsible for Exercise-Induced Ischemia on Days with Elevated Air Pollution? The ULTRA Study

**DOI:** 10.1289/ehp.8578

**Published:** 2006-01-13

**Authors:** Timo Lanki, Jeroen J. de Hartog, Joachim Heinrich, Gerard Hoek, Nicole A.H. Janssen, Annette Peters, Matthias Stölzel, Kirsi L. Timonen, Marko Vallius, Esko Vanninen, Juha Pekkanen

**Affiliations:** 1 Environmental Epidemiology Unit, National Public Health Institute, Kuopio, Finland; 2 Environmental and Occupational Health Division, Institute for Risk Assessment Sciences, Utrecht University, the Netherlands; 3 Institute of Epidemiology, GSF-National Research Center for Environment and Health, Neuherberg, Germany; 4 Center for Environmental Health Research, National Institute for Public Health and the Environment, Bilthoven, the Netherlands; 5 Department of Clinical Physiology and Nuclear Medicine, Kuopio University Hospital, Kuopio, Finland; 6 University of Kuopio, Kuopio, Finland

**Keywords:** air pollution, cardiovascular disease, elements, soot, vehicle emission

## Abstract

Epidemiologic studies have shown that ambient particulate matter (PM) has adverse effects on cardiovascular health. Effective mitigation of the health effects requires identification of the most harmful PM sources. The objective of our study was to evaluate relative effects of fine PM [aerodynamic diameter ≤ 2.5 μm (PM_2.5_)] from different sources on exercise-induced ischemia. We collected daily outdoor PM_2.5_ samples between autumn 1998 and spring 1999 in Helsinki, Finland. The mass of PM_2.5_ was apportioned between five sources. Forty-five elderly nonsmoking persons with stable coronary heart disease visited a clinic biweekly for submaximal exercise testing, during which the occurrence of ST segment depressions was recorded. Levels of PM_2.5_ originating from local traffic and long-range transport were associated with ST segment depressions > 0.1 mV, with odds ratios at 2-day lag of 1.53 [95% confidence interval (CI), 1.19–1.97] and 1.11 (95% CI, 1.02–1.20) per 1 μg/m^3^, respectively. In multipollutant models, where we used indicator elements for sources instead of source-specific PM_2.5_, only absorbance (elemental carbon), an indicator of local traffic and other combustion, was associated with ST segment depressions. Our results suggest that the PM fraction originating from combustion processes, notably traffic, exacerbates ischemic heart diseases associated with PM mass.

For a long time, it was assumed that ambient particulate matter (PM) damages primarily the lungs (Dockery and Pope 1994; Pope 1989). Epidemiologic evidence accumulated during the last decade shows that daily PM levels are also associated with daily changes in mortality and hospitalizations due to cardiovascular diseases (Katsouyanni et al. 2001; Samet et al. 2000; Schwartz 1999; Zanobetti et al. 2000). Cohort studies suggest that cumulative effects of air pollution on cardiovascular health are even more important than acute effects (Pope et al. 2002, 2004). Although the relative risks associated with exposure to PM are small compared with some other cardiovascular risk factors, the attributable health effects may be enormous because of the ubiquitous nature of exposure. As an example, 6% of annual deaths has been assigned to PM air pollution in three European countries (Künzli et al. 2000). Consequently, the World Health Organization (WHO) in the 1990s included air pollution on the list of 10 major risks for death and disability (WHO 1996).

In general, air quality has markedly improved in developed countries during the last few decades. Unfortunately, no lower threshold has been observed for the effects of PM (Daniels et al. 2000), and further efforts to decrease the PM levels are increasingly costly. Cost-effective mitigation of health effects requires regulation of primarily those PM sources that are responsible for the effects. However, the constituents and characteristics of PM responsible for the observed cardiovascular effects are unknown. Toxicologic studies suggest the importance of transition metals (Goldsmith et al. 1998; Pagan et al. 2003), organic carbon compounds (Bonvallot et al. 2001; Urch et al. 2004), endotoxins (Long et al. 2001; Osornio-Vargas et al. 2003), and the smallest PM fraction, ultrafine particles (UF; < 0.1 μm in diameter) (Brown et al. 2003; Li et al. 2003). The few epidemiologic studies that have compared the relative effects of different sources on cardiovascular health suggest that combustion particles are more important than crustal particles (Laden et al. 2000; Mar et al. 2000; Tsai et al. 2000).

The occurrence of ST segment depressions during stress tests indicates myocardial ischemia [American College of Cardiology/American Heart Association (ACC/AHA) 1997]. We have previously shown in a panel of coronary heart disease patients that increases in the daily levels of UF and fine PM [aerodynamic diameter ≤ 2.5 μm (PM_2.5_)] are associated with increased risk of ST segment depressions in Helsinki, Finland (Pekkanen et al. 2002). Our results provided an explanation for the associations observed in epidemiologic studies between ambient PM and hospitalizations and death due to ischemic heart diseases. We recently apportioned PM_2.5_ measurements of the earlier study between five major sources (Vallius et al. 2003). Using these data, we now evaluate the relative, short-term effects of PM_2.5_ from different sources on myocardial ischemia.

## Materials and Methods

### Study population and protocol.

The ULTRA (Exposure and Risk Assessment for Fine and Ultrafine Particles in Ambient Air) study was conducted in Amsterdam, the Netherlands, Erfurt, Germany, and Helsinki, Finland, between autumn 1998 and spring 1999. In each city, we followed panels of elderly subjects with stable coronary heart disease for 6 months with biweekly clinic visits and daily symptom and medication diaries. At the same time, levels of PM and gaseous air pollutants were monitored at fixed outdoor monitoring sites. We conducted all methods according to standard operating procedures (Pekkanen et al. 2000).

The main inclusion criteria for the study were a self-report on a doctor-diagnosed coronary artery disease and being a nonsmoker and at least 50 years of age. The study subjects were characterized by a questionnaire and recording of a 12-lead standard resting electrocardiogram. For each subject, the clinic visit was scheduled for the same weekday and time of day. At every visit, ambulatory electrocardiogram was recorded during a standardized protocol, which included 6-min submaximal exercise with a bicycle ergometer (Pekkanen et al. 2002).

Ethical committees in each country had approved the study protocol. All subjects gave a written informed consent before the study.

### Recording of ST segment depressions.

We have described elsewhere the methodology used for the recording of ST segment depressions in detail (Pekkanen et al. 2002). Briefly, the ambulatory electrocardiograms were recorded with Oxford MR-63 tape recorders (Oxford Instruments, Abington, UK), and the recordings were analyzed with the Oxford Medilog Excel II system (V7.5, Oxford Instruments) in one core laboratory. ST depressions were measured at 63 msec after J-point. Only ST depressions having their start time during the exercise test were considered.

We used two end points in the present analyses: ST depressions > 0.1 mV regardless of the direction of the ST slope (Rijneke et al. 1980), and ST depressions > 0.1 mV with horizontal or downward slope (i.e., stricter criteria) (ACC/AHA 1997). The present analyses are limited to Helsinki, because there were only seven ST segment depressions > 0.1 mV in Amsterdam and five in Erfurt (Pekkanen et al. 2002). This was mainly due to the low heart rates achieved during the exercise tests. The low heart rates were mostly attributable to the fact that the study was done in the community setting and the fieldworkers were very cautious in performing the exercise tests among coronary patients.

### Exposure monitoring.

In Helsinki, all study subjects lived within a 5-km radius of the fixed outdoor PM_2.5_ monitoring site. We collected daily noon-to-noon PM_2.5_ filter samples with single-stage Harvard impactors, 1 November 1998 to 30 April 1999. PM_2.5_ concentrations were determined gravimetrically. After weighing, blackness of filters was measured and transformed into absorption coefficients [i.e., absorbance (ABS)]. ABS is correlated with elemental carbon content of urban PM (Cyrys et al. 2003; Janssen et al. 2001). We have reported the laboratory methods and quality-control results elsewhere (Brunekreef et al. 2005; Janssen et al. 2001).

All PM_2.5_ filter samples were analyzed for elemental composition with energy-dispersive X-ray fluorescence spectrometry. Automated Tracor Spectrace 5000 system (Tracor X-ray, Austin, TX, U.S.) was used for analyses in the Department of Chemistry, University of Antwerp (Belgium). The accuracy varied between 1 and 28% depending on the element and its concentration on the filter. Eighteen elements were analyzed. Details of the elemental analyses have been reported earlier (Brunekreef et al. 2005; Janssen et al. 2005).

Number counts of UF were measured with an electrical aerosol spectrometer developed in the Institute of Environmental Physics of the University of Tartu, Estonia (Tammet et al. 2002). The Helsinki Metropolitan Area Council (Helsinki, Finland) provided the data on daily average temperature, and the Finnish Meteorological Institute (Helsinki, Finland) provided the data on relative humidity.

### Data analysis.

We used principal component analysis and multivariate linear regression to apportion PM_2.5_ mass between different sources. We identified source categories by examining the loadings of elements and other variables on the varimax (orthogonal) rotated principal components. We obtained estimates of daily source-specific PM_2.5_ concentrations by regressing the measured PM_2.5_ concentrations on absolute principal component scores (Thurston and Spengler 1985). Besides components of PM_2.5_ (elemental concentrations and ABS), daily data on UF and accumulation mode PM (diameter 0.1–1.0 μm), nitrogen dioxide, and sulfur dioxide were used to identify sources. We excluded 4 days from the elemental data because of outliers, leaving 164 days for analyses. Five main source categories were identified: local traffic (with contribution from other local combustion), long-range–transported air pollution, crustal source, oil combustion, and salt source. The source apportionment methodology and results have been previously published (Vallius et al. 2003).

Air pollution concentrations of 0- to 3-day lags were evaluated. Lag 0 concentration was defined as the 24-hr average from noon the previous day to noon of the day of the clinic visit; lag 1 concentration as the average concentration of the previous 24 hr, and so on. A basic confounder model was built for ST segment depressions > 0.1 mV without including PM, and the same model was used for the horizontal- or downward-sloping ST segment depressions > 0.1 mV. Loess functions were used to explore shapes and lags (0–3) of the covariates in S-Plus 2000 software (MathSoft Inc., Seattle, Washington, USA) (Hastie and Tibshirani 1990). Criteria for building the basic model were Akaike’s Information Criterion (Akaike 1974) and covariate-response plots. The protocol of statistical analyses is available at the ULTRA study web-site (http://www.ktl.fi/ultra). Because of the recently raised questions on the usability of generalized additive models in S-Plus for air pollution research (Dominici et al. 2002), we conducted final analyses in the statistical package R (R Project 2006) using logistic regression (R-GAM function) with penalized splines (Eilers and Marx 1996; Wood 2001). The basic model included a dummy for each subject; linear terms for time trend, temperature (lag 3), and relative humidity (lag 3); and a penalized spline (df = 3) for change in heart rate during the exercise test.

As a sensitivity analysis, we evaluated the effect of extreme concentrations on results. For each PM_2.5_ source, we calculated an outlier concentration corresponding to three times the interquartile range of each lag and excluded all observations higher than the lowest of these outlier concentrations from all the lags.

Varimax rotation used in principal component analysis forces the daily source-specific PM_2.5_ concentrations to be noncorrelating (Thurston and Spengler 1985), which does not always reflect reality. To allow correlation, we also constructed multipollutant models where indicator elements for sources were used instead of source-specific mass. For every source, the element with the highest correlation with source-specific mass was chosen as the indicator.

Finally, we evaluated whether potentially toxic elements, not among the indicator elements, would be associated with ST segment depressions. Various two-pollutant models were also constructed using individual elements and UF, but gaseous pollutants were not included in the models. Gaseous pollutants measured at a fixed outdoor monitoring site have been observed to reflect better exposures to PM_2.5_ than to gases themselves (Sarnat et al. 2005). Thus, interpretation of the models would have been difficult.

## Results

There were 417 exercise tests performed during 511 visits among 47 subjects. Of the 417 exercise tests, we excluded 67 because of substandard quality of the recording and eight because of missing data on covariates, leaving 342 exercise tests for analyses. There were altogether 45 study subjects in the final analyses; 24 of them were male. Mean (± SD) age of the subjects was 68.2 ± 6.5 years. Twenty-three of the subjects had had coronary bypass surgery or percutaneous transluminal coronary angioplasty, 27 had history of myocardial infarction, and 31 used beta-blockers. Details of the study panel have been published previously (Pekkanen et al. 2002).

During the study, 164 valid outdoor samples were collected. Thirteen elements [potassium (K), vanadium (V), manganese (Mn), copper (Cu), zinc (Zn), lead (Pb), aluminum (Al), silicon (Si), sulfur (S), chlorine (Cl), calcium (Ca), iron (Fe), nickel (Ni)] were detectable in > 50% of samples, and the rest were excluded from analyses. In addition, Pb and Al were left out of the present analyses because of low precision and reliability (Janssen et al. 2005). However, these elements were used in source apportionment, because even less precisely detected elements can improve the apportionment of PM between sources (Vallius et al. 2003).

Descriptive statistics for components of PM_2.5_ and UF are presented in Table 1. Sulfur was the most abundant element. Half of mass was apportioned to sulfur-rich long-range–transported PM_2.5_.

Many of the elements were highly correlated (Table 2). PM_2.5_ was highly correlated with K, S, Zn, and ABS but not with UF. The highest correlation coefficients between elements and source-specific PM_2.5_ were found between Si and crustal PM_2.5_, S and long-range–transported PM_2.5_, Ni, and V and PM_2.5_ from oil combustion, Cl and PM_2.5_ from salt source (salt source also characterized by high Pb concentrations), and ABS and traffic-originating PM_2.5_.

Median (minimum) daily temperature and relative humidity during the study period were −0.44°C (−23.3) and 87.6% (51.1), respectively. Temperature was negatively correlated with PM_2.5_ from local traffic (*r* = −0.33) and oil combustion (*r* = −0.35), and relative humidity with PM_2.5_ from oil combustion (*r* = −0.36) and crustal source (*r* = −0.55).

Associations between levels of source-specific PM_2.5_ and the occurrence of ST segment depressions are presented in Table 3. PM_2.5_ originating from local traffic as well as long-range–transported PM_2.5_ were associated with ST segment depressions > 0.1 mV. In addition, when the stricter criterion for ST segment depressions was used, a suggestive association of PM_2.5_ originating from oil combustion emerged. The highest observed odds ratios (ORs) were for PM_2.5_ from crustal source and from salt source at lag 3, but the associations were not consistent or statistically significant.

Exclusion of extreme concentrations (at maximum 13 measurements per lag) from source-specific PM_2.5_ changed the results only for oil combustion. The OR at lag 2 fell below 1, and for the strict criteria for ST segment depression, the association got weaker.

In a multipollutant model that included indicator elements for all sources, only ABS, a marker for local traffic, was associated with ST segment depressions (Table 4). The confidence intervals (CIs) were rather wide, and wider than in single-pollutants models (data not shown), obviously because of inclusion of several correlating variables in the same model.

In the single-pollutant models most of the individual elements—Cu, Fe, K, Mn, Ni, S, and Zn—were significantly (*p* < 0.05) associated with ST segment depressions > 0.1 mV at lag 2 (data not shown), possibly due to high intercorrelations. However, when potentially toxic elements (Cu, Fe, Zn, V) were included in two-pollutant models together with ABS, none of them was associated with ST segment depressions. In all two-pollutant models, statistically significant association between ABS and ST segment depressions > 0.1 mV remained at lag 2, and ORs for ABS varied only slightly: from 3.93 to 5.03 (data not shown).

**Figure 1:**
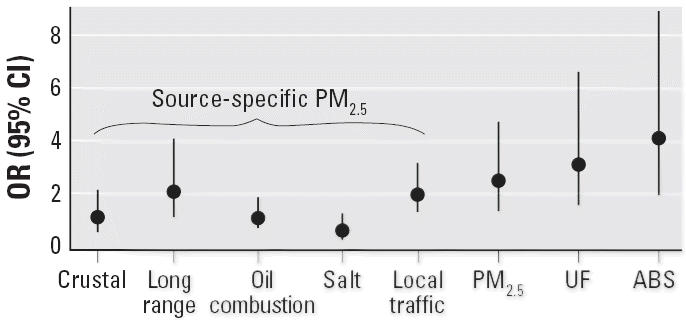
ORs and 95% CIs for 2-day lag for the association of source-specific PM_2.5_, (total) PM_2.5_, UF, and PM_2.5_ ABS with the occurrence of ST segment depressions > 0.1 mV. ORs were calculated for an increase of interquartile range of pollutant.

Interquartile range of long-range–transported PM_2.5_ was much higher than interquartile ranges of other source-specific masses (Table 1). We therefore also calculated ORs at lag 2 per interquartile range of source-specific PM_2.5_ for ST segment depressions > 0.1 mV (Figure 1). For comparison, ORs for the association of (total) PM_2.5_, UF, and ABS are also presented.

Finally, we tested whether the association of UF with ST segment depressions > 0.1 mV is robust to adjustment for ABS. The effect of ABS remained, but UF was no more significantly associated with ST segment depressions at lag 2. OR (95% CI) for ABS was 3.44 (1.25–9.50), and for UF, 1.38 (0.53–3.61).

## Discussion

Exposure to air pollution, especially PM, has been linked to exacerbations of ischemic and arrhythmic cardiac diseases and congestive heart failure (Kwon et al. 2001; Le Tertre et al. 2002; Peters et al. 2000; Zanobetti et al. 2000) and triggering of the onset of myocardial infarction (Peters et al. 2001, 2004). Promotion of atherosclerosis is likely to be involved in the chronic pathways of air pollution effect (Brook et al. 2004). The plausibility of a causal association is increased by the established association between passive smoking and heart disease (Barnoya and Glantz 2004). Our research team has previously published evidence on the association between PM_2.5_ and myocardial ischemia, as indicated by the occurrence of exercise-induced ST segment depressions among patients with coronary heart disease (Pekkanen et al. 2002). The observed association provided a plausible biologic link between levels of PM air pollution and adverse cardiac outcomes (Verrier et al. 2002). The same data were used in the present study.

Ambient PM is a very heterogeneous mixture of organic and inorganic components from multiple sources, and it is unlikely that all components have similar effects on the cardiovascular system. Our present results suggest that the composition of PM_2.5_ originating from local traffic is the most toxic, because the OR per microgram was high and the association was statistically significant. However, the effect of long-range–transported PM_2.5_ was comparable with the effect of traffic-originating PM_2.5_ when the estimate was calculated per measured interquartile range of the pollutant. This is caused by greater daily variability in the levels of long-range–transported PM_2.5_ and implies that by reducing high-concentration days of either of the sources, equal benefits are attainable. Associations between other sources and ST segment depressions were less consistent and not statistically significant, although high ORs were observed. PM_2.5_ originating from local traffic correlated highly with UF (*r* = 0.79), whereas long-range–transported PM_2.5_ correlated highly with PM_2.5_ (*r* = 0.82). Considering this, present results are in line with our previous study (Pekkanen et al. 2002), where we found levels of UF and PM_2.5_ to be independently associated with ST segment depressions.

There are few earlier studies that have compared health effects of PM_2.5_ from different sources, and all of them are mortality studies in the United States. The studies suggest effects of PM_2.5_ from a variety of combustion sources, and fewer effects of soil-originating PM_2.5_. In a time-series study of six eastern U.S. cities (Laden et al. 2000), only PM_2.5_ from traffic was weakly associated with increased daily mortality from ischemic heart disease, whereas both traffic and coal combustion were associated with increased total mortality. There was also a suggestion of a positive effect of oil-combustion–originating PM_2.5_ on total mortality in the cities where the source was identified. In Phoenix (Mar et al. 2000), cardiovascular mortality was associated with PM_2.5_ from traffic, biomass combustion, and regional pollution (indicated by sulfate). Total mortality was associated positively, although inconsistently, with regional sulfate. At three sites in New Jersey (Tsai et al. 2000), both total and cardiorespiratory mortality were associated with several sources of inhalable PM (aerodynamic diameter < 15 μm) including, depending on site, traffic and oil combustion, industry, and sulfate aerosol.

The source-apportionment method as used in our study yields noncorrelating source-specific PM_2.5_ concentrations. In an alternative approach, we constructed multipollutant models where indicators of sources were used instead of source-specific masses, and thus sources were allowed to correlate. In these models, only ABS (measure of elemental carbon), the chosen indicator for local traffic, was associated with ST segment depressions, whereas the effect of long-range PM_2.5_, as represented by sulfur, disappeared. ABS correlated in our study both with local traffic and long-range–transported PM_2.5_ (*r* = 0.74 and *r* = 0.46, respectively). Thus, results suggest that the effect of long-range PM_2.5_ is more related with carbon products than with secondary sulfate. ABS is associated with a variety of combustion processes, but ambient concentrations are mostly affected by diesel particles (Gray and Cass 1998).

It has been suggested that transition metals and/or organic carbon compounds adsorbed onto the elemental carbon core formed in incomplete combustion are responsible for the cellular changes associated with PM (Chin et al. 1998; Obot et al. 2002). Capability to induce oxidative stress in lungs is common to transition metals and organic carbon compounds (Kelly 2003). In the present study, most of the elements were associated with ST segment depressions, probably due to high intercorrelations. We did not find evidence for an effect of any of the potentially toxic transition metals when adjusting for ABS.

Recently, some toxicologic studies have suggested that the effects of UF on human health would be due to organic chemicals (Li et al. 2003; Xia et al. 2004). On the other hand, the composition of UF might not be decisive, but even inert PM in the UF size range seems to be capable of inducing oxidative stress because of great number, large surface area, and high penetration (Brown et al. 2003; Donaldson et al. 2001). When we adjusted UF for ABS, the association of UF with ST depressions weakened. Two-pollutant models of correlating variables should be interpreted with caution, but the results suggest that carbon content of PM may be more important than size. Consequently, the apparently independent effects of PM_2.5_ and UF that we observed previously could be explained by considering the two pollutants as indicators of two combustion sources with different temporal variation—long-range transport and local traffic.

We have previously shown in this same study population that outdoor concentrations of ABS correlate longitudinally (the relevant measure in time-series studies) highly with personal exposure, even better than PM_2.5_ (Janssen et al. 2000). There is a general lack of such information for UF, and it is possible that central outdoor measurements reflect worse day-today changes in exposure to UF than to ABS (Pekkanen and Kulmala 2004). This could weaken the association of UF with ST segment depressions in the two-pollutant model. However, most of the study participants lived very close (within 2 km) to the central monitoring site, which is likely to improve not only spatial but also longitudinal correlation.

PM can increase cardiovascular disease risk through several pathways, which have different lags. It is hypothesized that the local inflammation in lungs caused by PM starts a cascade of events that leads to systemic inflammation as indicated by increased levels of inflammatory mediators in blood (Brook et al. 2004). The systemic inflammation in turn might transfer the myocardium and myocardial circulation into a state that is vulnerable to various triggers of acute coronary syndrome (Muller et al. 1994). In our study, the effect of ABS on ST segment depressions was delayed, with strongest association observed at a 2-day lag, which is in agreement with systemic inflammation. There is evidence that PM can also trigger acute events. PM_2.5_ has been observed to increase risk of myocardial infarction both several hours and several days after exposure (Peters et al. 2001). In a recent study, an association was found between exposure to traffic and the onset of myocardial infarction within 1 hr afterward (Peters et al. 2004). Chronic exposure to traffic-related air pollution has also been associated with adverse effects on health (Finkelstein et al. 2004; Gauderman et al. 2004; Hoek et al. 2002).

Low numbers posed two major limitations in our study. Low number of study subjects calls for caution when comparing the magnitudes of odd ratios. Modest number of elements available for analyses leaves room for speculations about unidentified harmful constituents. Above all, we were not able to analyze organic compounds. Newer, more sophisticated methods of source apportionment could also be used in the future. However, different source-apportionment methods usually produce similar main sources, although, for example, traffic is a more difficult source because of lack of unique elemental tracers (Thurston et al. 2005). We used UF and gaseous pollutants in the source apportionment, which clearly improved the results (Vallius et al. 2003). No method totally bypasses the fact that meteorology drives the concentrations of pollutants from all sources into the same direction. In our case, local traffic partly included emissions from other, nonspecified local combustion processes due to common meteorology. In any case, any source-specific PM_2.5_ in our study should be considered merely as a marker of a harmful factor in PM; causal factors are still to be identified.

An unavoidable limitation in the study was the exclusion of summer time, because the elderly in Helsinki tend to spend most of the summer at summer cottages in the countryside. It is difficult to say whether the effect estimates would have been higher or lower during summer than during the cooler seasons. Exposure to ambient PM is probably higher during summer because of more frequent opening of windows at home and more frequent engagement in outdoor activities. On the other hand, cold temperatures are associated with increased rate of ischemic events (Barnet et al. 2005), and there is some evidence that the effects of air pollution on ischemic heart diseases are higher during the cool season (Wong et al. 2002).

Airborne PM has conventionally been measured and regulated based primarily on mass. Effective reduction of health effects associated with mass of PM requires identification of the most harmful PM sources. Our results suggest that the fraction of PM originating from combustion processes, notably traffic, may be responsible for the observed effect of PM air pollution on ischemic heart diseases.

## Figures and Tables

**Table 1:** Descriptive statistics for source-specific mass, elements, and ABS of PM_2.5_, and UF (*n* = 164).

	Average	25th percentile	Median	75th percentile	Maximum
Mass (μg/m^3^)					
Crustal	0.6	0.0	0.4	1.1	5.3
Long-range transported	6.4	2.2	5.5	9.8	26.5
Oil combustion	1.6	0.6	1.3	2.3	12.2
Salt	0.9	0.3	0.8	1.2	5.9
Local traffic	2.9	1.7	2.5	3.4	12.0
Total	12.8	8.3	10.6	15.9	39.8
Elements (ng/m^3^)					
Ca	38.5	22.7	32.3	47.3	154.4
Cl	98.9	8. 1	36.4	102.0	1556.0
Cu	1.9	0.6	1.6	2.8	8.9
Fe	75.1	39.3	66.7	100.3	297.0
K	108.2	60.0	93.1	141.0	540.3
Mn	4.2	2.2	3.4	5.2	14.5
Ni	3.4	1.7	2.9	4.4	18.7
S	1540.1	839.3	1383.9	2084.5	4296.6
Si	96.0	17.2	60.3	135.5	662.4
V	7.5	3.2	6.6	9.8	26.8
Zn	20.5	11.3	16.8	25.1	75.3
ABS (m^−1^ × 10^−5^)	2.0	1.4	1.9	2.5	4.9
UF (cm^−3^)	16,900	11,000	14,700	20,500	50,300

**Table 2:** Spearman’s correlation coefficients between elements of PM_2.5_, UF, and source-specific PM_2.5_ (*n* = 164).

	PM_2.5_	ABS	Ca	Cl	Cu	Fe	K	Mn	Ni	S	Si	V	Zn	UF
ABS	0.70													
Ca	0.17	0.30												
Cl	−0.03	0.02	0.11											
Cu	0.42	0.68	0.42	−0.05										
Fe	0.38	0.59	0.69	−0.18	0.71									
K	0.84	0.73	0.40	−0.10	0.52	0.56								
Mn	0.49	0.65	0.48	−0.17	0.65	0.79	0.60							
Ni	0.54	0.52	0.37	−0.07	0.39	0.41	0.59	0.13						
S	0.85	0.45	0.01	−0.28	0.23	0.21	0.70	0.37	0.43					
Si	0.10	0.29	0.71	−0.20	0.45	0.79	0.34	0.54	0.29	−0.26				
V	0.59	0.42	0.31	−0.04	0.21	0.29	0.57	0.36	0.88	0.52	0.18			
Zn	0.77	0.80	0.29	−0.10	0.66	0.59	0.84	0.73	0.59	0.62	0.30	0.53		
UF	0.13	0.57	0.39	−0.11	0.48	0.47	0.24	0.45	0.33	−0.05	0.36	0.16	0.34	
Source-specific PM_2.5_														
Crustal	−0.01	0.14	0.80	−0.08	0.36	0.72	0.25	0.49	0.21	−0.12	0.88	0.12	0.19	0.23
Long-range transported	0.82	0.46	−0.06	−0.18	0.26	0.20	0.70	0.38	0.27	0.90	−0.06	0.38	0.64	−0.19
Oil combustion	0.35	0.24	0.17	−0.12	0.06	0.10	0.37	0.20	0.86	0.35	0.11	0.86	0.34	0.20
Salt	0.19	0.07	0.19	0.54	−0.03	−0.17	0.11	−0.11	0.11	0.03	−0.13	0.15	0.07	−0.04
Local traffic	0.26	0.74	0.20	0.04	0.71	0.47	0.30	0.54	0.21	0.02	0.20	0.04	0.50	0.79

All correlations > 0.17 or < −0.17 are statistically significant at *p* < 0.05.

**Table 3:** Adjusted ORs^a^ between daily source-specific PM_2.5_ concentrations and the occurrence of ST segment depressions.

		> 0.1 mV (*n* = 62)^b^		> 0.1 mV + slope (*n* = 46)^c^	
Source-specific PM_2.5_	Lag	OR	95% CI	OR	95% CI
Crustal	Lag 0	0.80	0.47–1.36	0.76	0.42–1.35
	Lag 1	0.66	0.40–1.10	0.41	0.22–0.79
	Lag 2	1.18	0.68–2.06	1.17	0.65–2.09
	Lag 3	1.87	0.85–4.09	1.60	0.72–3.59
Long-range transport	Lag 0	0.94	0.84–1.05	0.98	0.86–1.10
	Lag 1	1.00	0.92–1.08	1.03	0.95–1.12
	Lag 2	1.11	1.02–1.20	1.11	1.02–1.21
	Lag 3	1.06	0.95–1.18	1.02	0.95–1.10
Oil combustion	Lag 0	0.87	0.57–1.32	0.95	0.61–1.49
	Lag 1	1.04	0.75–1.45	1.13	0.76–1.68
	Lag 2	1.10	0.83–1.46	1.33	0.98–1.80
	Lag 3	1.12	0.79–1.58	1.29	0.90–1.86
Salt	Lag 0	1.03	0.57–1.85	1.15	0.56–2.38
	Lag 1	0.72	0.37–1.40	0.90	0.44–1.81
	Lag 2	0.66	0.31–1.40	1.39	0.63–3.08
	Lag 3	1.55	0.83–2.89	1.93	1.00–3.72
Local traffic	Lag 0	0.91	0.69–1.21	0.89	0.64–1.23
	Lag 1	1.22	0.88–1.69	1.21	0.86–1.71
	Lag 2	1.53	1.19–1.97	1.37	1.03–1.83
	Lag 3	0.98	0.78–1.23	1.03	0.80–1.32

ORs calculated for an increase of 1 μg/m^3^ in exposure. Number of measurements = 312 for lag 0, 322 for lag 1, 314 for lag 2, and 311 for lag 3.

a The statistical model included the source-specific PM_2.5_ concentration; a dummy for each subject; linear terms for time trend, temperature (lag 3), and relative humidity (lag 3); and a penalized spline (df = 3) for change in heart rate during exercise test.

b ST segment depression > 0.1 mV; *n* = number of events at minimum (lag 0).

c ST segment depressions > 0.1 mV with horizontal or downward slope; *n* = number of events at minimum (lag 0).

**Table 4:** ORs for the association of indicator elements of PM_2.5_ sources with the occurrence of ST segment depressions in multipollutant models.^a^

		> 0.1 mV (*n* = 62)^b^		> 0.1mV + slope (*n* = 46)^c^	
Source indicator	Lag	OR	95% CI	OR	95% CI
Si (crustal)	Lag 0	0.73	0.39–1.38	0.67	0.33–1.36
	Lag 1	0.48	0.25–0.93	0.34	0.15–0.81
	Lag 2	0.78	0.35–1.71	0.81	0.33–2.00
	Lag 3	1.95	0.69–5.48	1.90	0.64–5.65
S (long-range transport)	Lag 0	0.70	0.25–1.95	0.84	0.29–2.47
	Lag 1	0.58	0.23–1.47	0.89	0.34–2.32
	Lag 2	1.08	0.44–2.63	1.36	0.54–3.45
	Lag 3	1.60	0.73–3.48	1.12	0.53–2.40
Ni (oil combustion)	Lag 0	0.78	0.30–2.04	1.10	0.36–3.37
	Lag 1	1.20	0.58–2.46	1.16	0.45–2.96
	Lag 2	1.15	0.61–2.18	1.64	0.84–3.20
	Lag 3	1.02	0.41–2.54	1.63	0.64–4.14
Cl (salt)	Lag 0	1.03	0.79–1.34	1.13	0.80–1.62
	Lag 1	0.88	0.56–1.38	0.99	0.58–1.68
	Lag 2	1.02	0.62–1.69	1.55	0.87–2.76
	Lag 3	1.27	0.85–1.91	1.45	0.94–2.25
ABS (local traffic)	Lag 0	0.92	0.36–2.37	0.74	0.25–2.23
	Lag 1	1.83	0.73–4.59	1.76	0.62–5.00
	Lag 2	4.46	1.69–11.79	4.86	1.55–15.26
	Lag 3	0.92	0.40–2.12	0.97	0.39–2.41

ORs calculated for an increase of interquartile range of pollutant. Number of measurements = 312 for lag 0, 322 for lag 1, 314 for lag 2, and 311 for lag 3.

a The statistical model included all five indicator elements, a dummy for each subject, linear terms for time trend, temperature (lag 3) and relative humidity (lag 3), and a penalized spline (df = 3) for heart rate during exercise test.

b ST segment depression > 0.1 mV; *n* = number of events at minimum (lag 0).

c ST segment depressions > 0.1 mV with horizontal or downward slope; *n* = number of events at minimum (lag 0).
